# 

**Published:** 1916-12-16

**Authors:** 


### Further Patriotism in British Hospitals.

IN our notice of St. Thomas's Hospital last week we regret the number of past and present students who have died on service was given as 228 instead of 29.

### Passing Events and Topics.

### Colonel Lister's Estate.

The late Colonel A. H. Lister, M.D., C.M.G., lecturer in clinical medicine at Aberdeen University, and a nephew of the late Lord Lister, left personalty amounting to £31,720.

### Manchester Royal Infirmary.

Two vacancies on the Board of the Manchester Royal Infirmary, caused by the death of Brigadier-General Noel-Lee and the resignation of Mr. Neville Clegg, have been filled by the appointment of Dr. E. Craven Moore, F.R.C.P., and Mr. Assheton Clegg.

### Allington Manor Sanatorium.

This sanatorium at Eastleigh is maintained by the Allies Relief Committee for the benefit of Belgian soldiers suffering from tubercular trouble, and has been open since August 1915. Thirty-one cases have been received, and half of the patients show improvement or have been cured.

### Medical Appointments.

Dr. William Ewen Reid, M.B., Ch.B. Aberdeen, has been appointed by the Bermondsey Board of Guardians non-resident assistant medical officer and deputy to the chief medical officer and medical superintendent of the infirmary, at a salary of £400 a year, rising by annual increments of £25 to a maximum of £450, together with an allowance of £50 per annum in lieu of residence.

Professor G. Sims Woodhead, M.A., M.D., C.M., F.R.S.E., has been reappointed consultative bacteriological adviser to the Metropolitan Asylums Board at a salary of £105 per annum.

Dr. James Galloway, consulting physician for skin diseases at the Goldie Leigh Homes, who obtained leave of absence from the Metropolitan Asylums Board to enable him to undertake work in connection with the troops in France, has returned to his duties after an absence of seven months.

Dr. William W. Kelly, M.B., B.C.L., B.A.O., has been appointed temporary assistant medical officer at the Lambeth Infirmary at a salary of seven guineas a week. Dr. Lambrinudi, who acted as *locum tenens*, has obtained permission to proceed to Salonica to join the Greek forces, and will leave the service of the Lambeth Board of Guardians as soon as the medical superintendent can replace him.

### Medical Examinations—Alleged Deception.

Mr. Snowden asked the Secretary of State for War if instructions could be issued that an applicant who has two conflicting classification cards, as results of examination on different dates, shall have the right to appeal to the Central Medical Board. Mr. Forster replied that the practice of going before several Boards has now been stopped; formerly it transpired that certain persons, in the desire to avoid service, were going before more than one Medical Board, sometimes after taking drugs. It, therefore, became a practice among military representatives to accept the opinion of the first Board as reliable.

### Matrons' and Sisters' Appointments.

Manchester, Elm Bank Red Cross Hospital, Eccles.—Miss Mabel Flower has been appointed sister. She was trained at Kendal County Hospital, and has since held various appointments.

Rawtenstall Auxiliary Military Hospital.—Miss Ethel V. Brew has been appointed sister. She was trained at Hope Hospital, Pendleton, where she has since been staff nurse and sister. She has also done private nursing.

**Note.—Particulars of Appointments for insertion in this column will be welcomed.**

**Fife and Kinross Board of Control.**—The admissions for the year ending May 15 to the Fife and Kinross District Asylum were 199, the largest admission rate on record, increased, probably, through the asylum at Bangour being taken over as a war hospital. Serious defects have been discovered in some new buildings recently constructed, and the remedying of the various defects have been considered by a special committee, whose recommendations have been carried out.

### Gifts and Bequests.

Mr. Edward Cottam, of Cardiff and Rotherham, has placed a gift of £1,000 at the disposal of the weekly board of the Rotherham Hospital.

Lord Llangattock, who died on Octoebr 31, left £100 to the Monmouth Hospital and Dispensary.

Mr. Bickerton Horner Deakin, Town Clerk of Monmouth, who died on June 24, left £500 to the Monmouth General Hospital and Dispensary for the children's ward, in memory of his " darling child, Lizzie " ; and he also left to the hospital a framed portrait of his child, desiring that the authorities should place a wreath of flowers on her grave each Palm Sunday and a wreath of holly each Christmas Day.

Mr. William Somerville Letten, of Kempthorne, Wellholme Road, Grimsby, who died on January 14, left £250 to the Grimsby District Hospital and £100 to the Moorfields Eye Hospital, London.

Mrs. Catherine Nash, of Eastbourne, has left £5,000 to the Middlesex Hospital, for a ward to be named the Robert George Nash Ward.

Amongst other bequests, Mr. Robert Tait, of Cheetham, Manchester, left £100 to the Manchester Royal Infirmary and £100 to the Salford Royal Hospital.

At the Italian Hospital, Queen Square, W.C., a bed has been endowed by Commendatore P. Polenghi, in memory of his wife.

Mrs. A. Sebag-Montefiore has endowed a bed at Ramsgate Hospital in memory of the late Captain Robert Sebag-Montefiore, who died of wounds received at Gallipoli.

On account of the profits of Dulac's Gift Book, a further payment of £526 has been made to the French Red Cross, to which a total of at least £1,500 will accrue from this source.

Ross Cottage Hospital has received a gift of a valuable *x*-ray equipment from Mrs. Arthur Foster, of Brockhampton Court, Ross.

A sum of £13,500 was left for charitable purposes by Mrs. E. K. Alston, of Kilmacolm, the larger part being devoted to Missions in India and connected with the Church of Scotland.

Mr. William Walter, of Erdington, has left £500 to the Queen's Hospital, Birmingham, £500 to the Blind Asylum at Edgbaston, and £1,250, for the endowment of a bed, to the General Hospital, Birmingham.

In commemoration of a visit by the Bishop of Manchester to the Nottingham General Hospital, when a special sermon was preached, Mr. W. G. Player, of Messrs. John Player and Sons, has endowed a bed at the hospital at a cost of £1,000.

Mr. Edwin Round, of Birmingham, has left £100 to the Guest Hospital, Dudley, and £50 each to the General Hospital, the Queen's Hospital, and the Children's Hospital at Birmingham, and the Children's Convalescent Home, Moseley Hill.

Rates of Subscription: Twelve months. 6/6; Six months, 4/-; Three months, 2/-; post free to any address in the United Kingdom. Abroad, Twelve months, 8/8; Six months, 5/-; Three months, 2/6, post free. The Hospital "Index" to Volume LX., April to September 1916, is now ready, price 2½d. per copy, post free. Address The Manager, "The Hospital," The Hospital Building, 28/29 Southampton Street, Strand, London, W.C.

### NATIONAL RELIEF FUND.

Treasurer—H.R.H. The Prince of Wales.

**Will every Reader of "THE HOSPITAL" send at least 1s.**

To H.R.H. The Prince of Wales,
Buckingham Palace, London

*I beg to enclose £ s. d. as a donation to the National Relief Fund.*

*Name* ..........................................

*Address* ..........................................

..........................................

The envelope containing this coupon need not be stamped.

